# Linkage to HIV care following diagnosis in the WHO European Region: A systematic review and meta-analysis, 2006-2017

**DOI:** 10.1371/journal.pone.0192403

**Published:** 2018-02-16

**Authors:** Sara Croxford, Zheng Yin, Fiona Burns, Andrew Copas, Katy Town, Sarika Desai, Andrew Skingsley, Valerie Delpech, on behalf of the OptTEST project

**Affiliations:** 1 Centre for Infectious Disease Surveillance and Control, Public Health England, London, United Kingdom; 2 Centre for Population Research in Sexual Health and HIV, Institute of Global Health, University College London, London, United Kingdom; 3 Royal Free London NHS Foundation Trust, London United Kingdom; Imperial College London, UNITED KINGDOM

## Abstract

**Background:**

Timely linkage to care after HIV diagnosis is crucial as delayed access can result in poor patient outcomes. The aim of this systematic review was to synthesise the evidence to achieve a better understanding of what proportion of patients are linked to care and what factors impact linkage.

**Methods:**

Systematic searches were run in six databases up to the end of February 2017. The grey literature was also reviewed. Inclusion criteria were: sample size ≥50 people (aged ≥15), from the WHO European Region, published 2006–2017 and in English. Linkage to care was defined as a patient seen for HIV care after diagnosis. Study selection, data extraction and quality assurance were performed by two independent reviewers. Random-effects meta-analysis was carried out to summarise linkage to care within three months of diagnosis.

**Results:**

Twenty-four studies were included; 22 presented linkage to care data and seven examined factors for linkage. Linkage among 89,006 people in 19 countries was captured. Meta-analysis, restricted to 12 studies and measuring prompt linkage within three months, gave a pooled estimate of 85% (95% CI: 75%-93%). Prompt linkage was higher in studies including only people in care (94%; 95% CI: 91%-97%) than in those of all new diagnoses (71%; 95% CI: 50%-87%). Heterogeneity was high across and within strata (>99%). Factors associated with delaying or not linking to care included: acquiring HIV through heterosexual contact/injecting drug use, younger age at diagnosis, lower levels of education, feeling well at diagnosis and diagnosis outside an STI clinic.

**Conclusion:**

Overall, linkage to care was high, though estimates were lower in studies with a high proportion of people who inject drugs. The high heterogeneity between studies made it challenging to synthesise findings. Studies should adopt a standardised definition with a three month cut-off to measure prompt linkage to care to ensure comparability.

## Introduction

Linking people who test HIV-positive to appropriate specialist services is a key step in the HIV patient pathway. Delayed linkage to HIV care is associated with delayed receipt of antiretroviral medications, faster disease progression and increased mortality.[1, 2] In addition to the impact on the health of the individual, engagement in HIV care plays an important public health role in reducing the onward transmission of HIV.[3]

Monitoring is crucial so that missed opportunities for linking patients to HIV services following diagnosis can be identified and gaps closed.[4] However, the variety of definitions of linkage to care applied in the literature makes it difficult to compare measurements across countries and studies.[5] A standard working definition of linkage to care would enable consistent monitoring of the quality of HIV care and patient clinical outcomes.

In May 2015, the World Health Organization (WHO) released strategic information guidelines in an effort to consolidate and prioritise key indicators to monitor national and global response of the health sector to HIV.[4] Linkage to HIV care was defined as the duration of time starting with HIV diagnosis and ending with enrolment in HIV care or treatment.

This definition was endorsed and further operationalised following consultation with European experts brought together by the European Centre for Disease Prevention and Control (ECDC) and the European Union (EU) co-funded OptTEST (Optimising testing and linkage to care for HIV in Europe) project.[6] A person was considered linked to care if seen for specialist HIV care after diagnosis, measured as the time between the HIV diagnosis date and first clinic attendance date/CD4 count date/viral load date/HIV treatment start date, depending on data availability. A time parameter was added to the WHO definition and prompt linkage was defined as the proportion of patients diagnosed with HIV linked to care within three months.

The aim of this systematic review was to synthesise the evidence to achieve a better understanding of linkage to care following HIV diagnosis in the WHO European Region. The specific objectives were to: i) estimate prompt linkage to care from studies which utilised the standard ECDC/OptTEST definition and ii) determine what factors impact linkage.

## Methods

A protocol was developed prior to commencement of the systematic review and published on PROSPERO, an international prospective register of systematic reviews.[7]

### Study identification

Searches were carried out on the 27th of February 2017 in Embase (Ovid 1974 –present), MEDLINE (Ovid MEDLINE(R) Epub Ahead of Print, In-Process & Other Non-Indexed Citations), PubMed, Cochrane Database of Systematic Reviews, PsycINFO (PsycINFO 1806 to February Week 4 2017) and Web of Science Core Collection. Database searches covered HIV, linkage/entry/referral to care and Europe. Specific search strings can be found in Tables A-F in S1 Appendix.

Conferences abstracts from the International AIDS Society conference (IAS), International AIDS conference (AIDS), European AIDS Clinical Society conference (EACS), HIV Drug Therapy conference Glasgow, HIV in Europe conference and the Conference on Retroviruses and Opportunistic Infections (CROI) were reviewed for relevant studies. In addition, the websites for the WHO, the Joint United Nations Programme on HIV/AIDS (UNAIDS) and ECDC were searched for relevant online reports.

### Study inclusion and exclusion criteria

To be included in the systematic review, studies had to be in English, set in the 53 countries of the WHO European Region, have a sample size of at least 50 people and be published between the 1^st^ of January 2006 and 27^th^ of February 2017. The 2006 date restriction reflects the release of the WHO patient monitoring guidelines for HIV care and antiretroviral therapy (ART), outlining essential minimum standard HIV care and ART patient monitoring data elements.[8]

Observational studies using data collected for surveillance or research purposes and qualitative studies including quantitative outcome data on linkage to care were included. Intervention evaluation studies were also included where linkage to care was reported.

Studies of people <15 years of age were excluded. Studies combining adults and paediatric/adolescent patients were included only if over 50% were aged ≥15.

The definition of linkage to care applied at full-text review stage was: a patient seen for HIV care (measured by first clinic attendance date/CD4 count/viral load measurement/treatment start date) after diagnosis. A CD4 count, viral load or evidence of treatment initiation after diagnosis was considered a proxy for entry into care.

### Study selection

Two independent reviewers screened titles and abstracts and assessed the eligibility of accepted studies through full-text review. Disagreement was resolved through consensus or independent adjudication by a third party. Reference lists of studies selected for inclusion were hand-searched with any relevant studies not previously identified, screened and full-texts reviewed.

For included studies that required further clarification regarding the reported data or definition of linkage, study authors were contacted by email. A maximum of two attempts to contact the corresponding and/or senior author were made. In some cases, data were updated by the authors.

### Data extraction and quality assessment

Data were extracted from the final list of included studies by one reviewer and all results checked by a second reviewer. Data were entered into a standardised data collection form on Microsoft Access 2010, capturing information on the publication, the study design, population, outcomes and risk of bias. Where the study included data from outside the review period (prior to 2006), only data on people diagnosed from 2006 onwards were extracted. Studies were categorised into geographical regions based on WHO/ECDC classifications.[9]

Quality assessment of the included peer-reviewed articles was carried out by two independent reviewers following established criteria, adapted to cover longitudinal study designs.[10]

### Statistical analysis

Heterogeneity was quantified using the Q statistic and I^2^ statistic; I^2^ values around 25%, 50% and 75% were taken to represent low, medium and high heterogeneity, respectively. A random effects model of single proportions with binomial exact confidence intervals (CI) was used to aggregate results for prompt linkage to care at three months. Proportions were stabilised using the Freeman–Tukey double arcsine transformation. For data not combined in meta-analyses, such as linkage to care at one month and six months, individual results were compared in descriptive analyses. Data were analysed separately based on care status of the study population. Specifically, whether the denominator for each study included: i) all new diagnoses, including those not linked to care or ii) only people in care with care information available (e.g. people with CD4 data). No population subgroup analyses were carried out (e.g. by risk group) due to the relatively small number of studies. There were so few published studies; publication bias could not be assessed using a funnel plot.

Risk factors for poor linkage to care could not be synthesised using meta-analysis given the variety of outcomes explored in the different studies (e.g. risk factors for those never linked, risk factors for delayed linkage etc.).

All statistical analyses were carried out using Stata v13.0 (College Station, Texas, USA).

## Results

### Study identification

**Fig 1:**
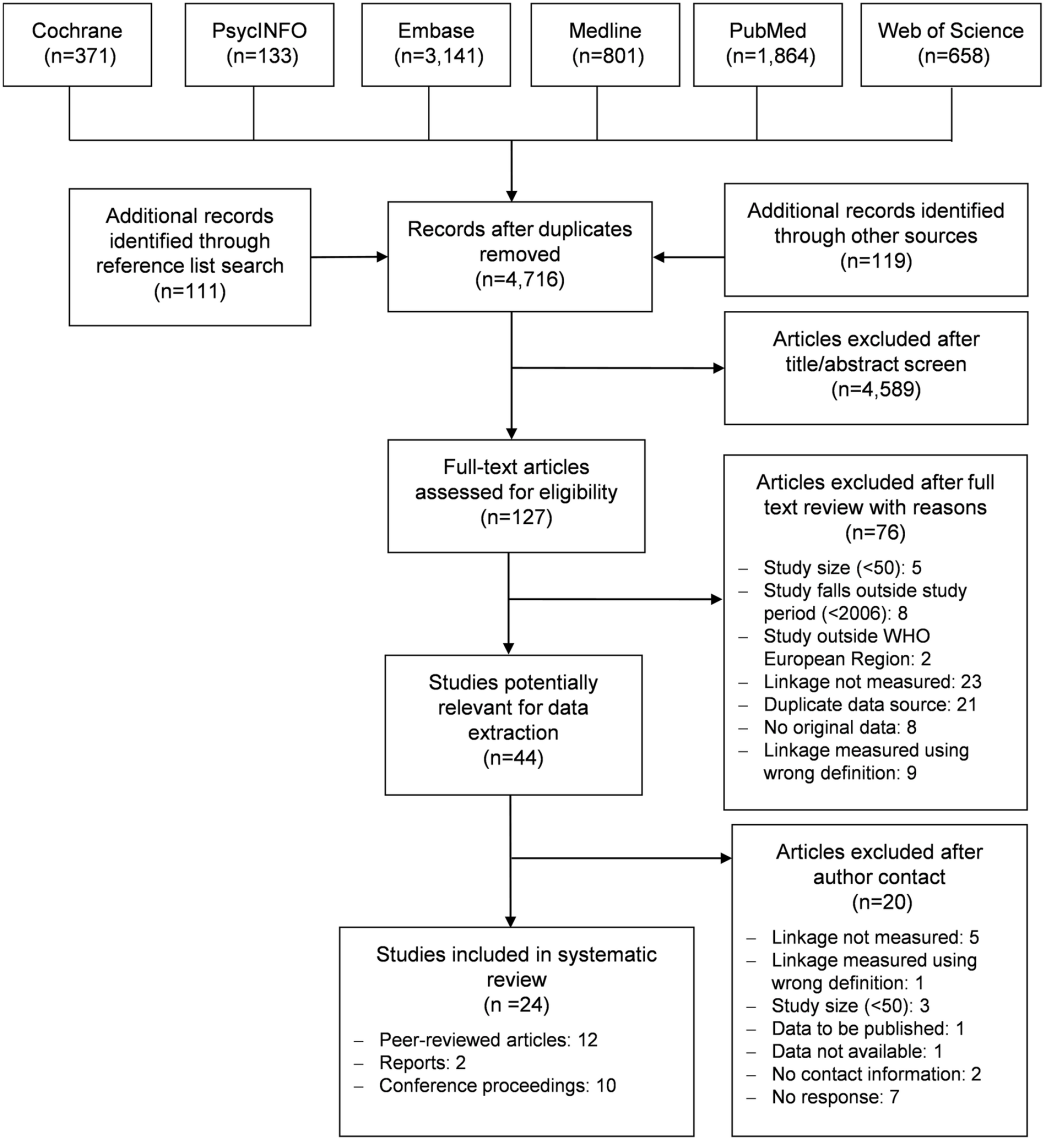
PRISMA (Preferred Reporting Items for Systematic Reviews and Meta-Analyses) flow diagram.

The database searches retrieved a total of 6,968 records (Fig 1). In addition, 119 abstracts were identified through the search of the grey literature. After deduplication, 4,707 unique records underwent title/abstract screening, 118 records were selected for full-text review and 42 studies were included. Reasons for exclusion can be seen in Fig 1. Reference lists from these included studies were scanned and 111 of the 566 references were deemed relevant. However, after deduplication, screening and full-text review, only two further studies were included.

Of the 44 included studies, 30 required clarification from authors. Two authors had no contact information available. Of the 28 authors contacted, 20 replied. Studies for which no reply was received were excluded. A further 11 studies were excluded after clarification. Reasons for rejection can be seen in Fig 1. In total, 24 articles met the eligibility criteria and were included in the review, 12 published articles [11–22], 10 conference proceedings [23–32] and two reports [33, 34].

### Description of linkage to care following diagnosis

All 24 studies presented data on linkage to care following diagnosis (Table 1); however, two studies [20, 31] have been excluded from the descriptive linkage to care analysis as more recent estimates were available from other studies using the same data source.[17, 34] These two studies have been included in the review as they identify risk factors for poor linkage.

**Table 1:** Characteristics of studies included in the systematic review (n = 24 studies).

Author, year	Country of study	Study period	Data source and setting of study	Study population	Sample size	Linkage to care outcome
Chernyshev*, 2017 [24]	Ukraine	Jan—Mar 2017	Community-based counselling and testing (CBVCT) testing sites in Kyiv and Odessa	Men who have sex with men (MSM) newly diagnosed with HIV through rapid testing between January and March 2017	65	First attendance for medical registration at the local AIDS centre after a positive result for rapid HIV testing
Croxford*, 2017 [26]	33 WHO European countries	2010–2014	European HIV surveillance	Adults (aged ≥15 years) diagnosed with HIV from 2010–2014 from WHO European countries that reported using the revised template—excluding those previously diagnosed or in care, deaths within 3 months of diagnosis and/or those with missing diagnosis/CD4 information	60,139	First attendance for specialist HIV care after diagnosis, as determined by the date of the first CD4 count after diagnosis
Freeman-Romilly, 2017 [14]	United Kingdom	2008–2012	Terrence Higgins Trust (THT) CBVCT with follow up at sexual health clinics	People who had received a reactive HIV test in a THT community clinic between 2008 and 2012	74	First attendance at an HIV clinic after diagnosis through community testing, using the date of the first reported CD4 as a proxy for care entry
Girometti, 2017 [15]	United Kingdom	May 2014- Oct 2015	56 Dean Street sexual health clinic in London	All individuals diagnosed with acute HIV infection between May 2014 and October 2015 at 56 Dean Street in London and starting ART at first appointment	113	Presence of at least one CD4+ T-cell count or viral load determination within 12 weeks of HIV diagnosis
del Campo*, 2016 [27]	Spain	2015–2016	Ramón y Cajal Hospital, Madrid	All first positive HIV results obtained in the Microbiology Laboratory Department of Ramón y Cajal Hospital from 01/01/2015 to 31/12/2016	112	First visit to the Infectious Service for HIV / AIDS after first HIV-positive serology
Elliot, 2016 [12]	United Kingdom	2012–2014	HIV home sampling service with follow-up at a London sexual health service	MSM testing positive through free home HIV sampling service (‘Dean Street at Home’ advertised via the same social media used to find sexual partners) confirmed and seen for care at Dean Street sexual health clinic	82	First attendance for HIV specialist care after diagnosis
Fernandez-Lopez, 2016 [13]	Denmark, Italy, Lithuania, Spain, Latvia	2016††	CBVCT sites across Europe	People with a reactive HIV test at CBVCT in 2016	112	Entry into health care or follow-up by an HIV specialist or in an HIV unit after diagnosis at a CBVCT facility
Kirwan*, 2016 [34]	United Kingdom	2015	National HIV surveillance	All adults (≥15 years of age at diagnosis) newly diagnosed with HIV in the UK in 2015 with a CD4 count after diagnosis reported.	5,149	Baseline CD4 count (conducted as part of initial assessment in care) after diagnosis
Kowalska, 2016 [18]	Poland	2010–2013	3 CBVCT sites in Central Poland	People who were diagnosed HIV-positive in CBVCTs between 1/1/2010 and 31/12/2013	232	First visit in the HIV clinic after testing HIV-positive
Neduzhko‡‡, 2016 [20]	Ukraine	Oct—Dec 2011	Odessa AIDS Centre	Patients (aged ≥18 years) recently registered for HIV care at Odessa AIDS centres able to provide a date of his or her positive HIV test result	200	Registered at an HIV care centre following diagnosis
Chkhartishvili*, 2015 [25]	Georgia	2008–2012	National HIV surveillance	Adult (aged ≥18 years) HIV-infected citizens of Georgia diagnosed in Georgia from 2008–2012	1,563	At least one documented clinical visit (CD4 cell count or HIV-1 viral load measurement) after diagnosis
Michie*, 2015 [28]	United Kingdom	Aug 2013- July 2014	Outpatient clinics in NHS Greater Glasgow and Clyde, Scotland	Outpatients in NHS Greater Glasgow and Clyde health board with a positive HIV result between 01/08/13–31/07/14	64	Seen by HIV physician after diagnosis
Raffo*, 2015 [29]	Spain	2009–2012,2014	Reference centre in infectious diseases Huelva Province	New diagnoses of HIV made between 2009 and 2012 in Huelva province compared to new diagnoses made in 2014	2009–2012: 176; 2014: 55	Patient went to a scheduled appointment to the HIV unit or if the patient has documented visit in another hospital after diagnosis
Van Beckhoven, 2015 [21]	Belgium	2007–2010	National HIV surveillance	Individuals diagnosed with HIV in Belgium between 2007 and 2010	4,117	At least one viral load or CD4 count recorded within 1 year of HIV diagnosis
Van Sighem*, 2015 [30]	Netherlands	2014††	ATHENA national HIV cohort	People diagnosed with HIV in the Netherlands in 2014 and registered in the ATHENA national observational HIV cohort	858	First attendance for HIV care and registration in the HIV clinical cohort after diagnosis
van Veen, 2015 [22]	Netherlands	Feb 2009—Jan 2012	STI clinics in Amsterdam, Rotterdam and Arnhem	From February 2009 until January 2012, all patients testing HIV-positive for the first time at STI clinics in Amsterdam, Rotterdam and Arnhem	310	First consultation at an HIV treatment centre after diagnosis
Zakowicz*, 2015 (32]	Russia, Ukraine, Georgia, Greece, Italy, Armenia, Ireland	Nov 21–28 2014	12 CBVCTs across Europe	People attending 12 community-based organisations during HIV testing week 2014 in 11 countries	138	Attendance at an HIV care and treatment facility two times for medical care following receipt of an HIV+ diagnosis or receipt of CD4 results
Cuzin, 2013 [11]	France	2006–2010‡	HIV reference centres in 8 regions	Patients with a first HIV diagnosis between 01/01/2006-31/12/2010 that had at least 1 medical encounter in 1 of 8 HIV reference centres in France	2,670	First HIV diagnosis during the study period that had at least 1 medical encounter in that HIV reference centre
Hall, 2013 [16]	Italy, Spain	2010	National HIV surveillance	People newly diagnosed with HIV in 7 regions of Spain in 2010 or in 18/21 regions of Italy in 2010 where CD4 data available	Italy: 3,245; Spain: 1,519	≥1 CD4 or viral load test within 3 months of HIV diagnosis
Kiriazova, 2013 [17]	Ukraine	2006–2010‡	Odessa AIDS Centre	Patients (aged ≥ 15 years) enrolled in HIV medical care at the Regional AIDS Centre in Odessa Region, Ukraine from 2006 to 2010	6,101	Enrolment in HIV care after diagnosis
Meulbroek, 2013 [19]	Spain	2007–2012	Barcelona Checkpoint CBVCT	HIV cases in MSM in Catalonia detected at BCN Checkpoint between 2009 and 2012	495	HIV unit referral of individuals newly diagnosed with HIV
Yin*‡‡, 2012 [31]	United Kingdom	2010	National HIV surveillance	Adults (aged ≥15 years) first diagnosed with HIV in 2010 in the UK reported as part of national HIV surveillance and with a CD4 count after diagnosis reported	5,662	First attendance for care of patients diagnosed with HIV, with the date of the first CD4 count as a proxy for care entry
Sprague*, 2011 [33]	Estonia, Moldova, Poland, Turkey, and Ukraine	2010–2011	Peer-administered survey**	People living with HIV in Estonia, Moldova, Poland, Turkey, and Ukraine who had accessed HIV testing services and received a diagnosis	Estonia: 87; Moldova: 403; Poland: 504; Turkey: 100; Ukraine: 1,500	Accessing care services (visit to a medical professional for one’s HIV infection) after receipt of an HIV diagnosis
Apea*, 2009 [23]	United Kingdom	2007	Homerton Hospital STI clinic in London	Patients newly diagnosed with HIV infection between 01/01/2007-31/12/2007	88	First attendance for care at an HIV clinic after diagnosis

*Conference proceedings or reports

**No information on where or how people were recruited

^††^ Data updated to more recent years after contact with authors

^‡^ Only included data from 2006 onwards

^‡‡^ Included in factor analysis only—linkage to care estimates are duplicates

The 22 included studies covered 19 of the 53 countries from the WHO European Region, with the most studies incorporating data from Western (Belgium, Denmark, France, Greece, Ireland, Italy, Netherlands, Spain and the United Kingdom (UK)) and Eastern Europe (Armenia, Estonia, Georgia, Latvia, Lithuania, Moldova, Russia and Ukraine). Only two studies presented data for Central Europe (Poland and Turkey). One study presented aggregate data for the WHO European Region but did not explicitly state which countries were covered.[26]

Data sources and the geographical coverage of data differed between studies (Table 1). Five studies measured linkage using national or European HIV surveillance data.[16, 21, 25, 26, 34] Six studies presented data on linkage following an HIV diagnosis from community-based voluntary counselling and testing (CBVCT) sites,[13, 14, 18, 19, 24, 32] while five studies described linkage from medical settings including sexually transmitted infection (STI) clinics and hospitals.[15, 22, 23, 27, 29] Four studies described retrospective entry of patients already attending HIV clinics.[11, 17, 28, 30] Elliot et al. looked at linkage into care following an HIV-positive self-sampling test [12] and Sprague et al. described linkage among people who had accessed HIV testing services, regardless of type.[33]

The linkage experience of a total of 89,006 people was captured across 22 studies. Study sizes ranged from 64 to 60,139, with <250 (range: 64 to 232) in 11 studies and ≥250 (range: 310 to 60,139) in the other 11. Over one third of studies covered a one-year period or less (36%; 8/22), but some covered several years (range: three months-five years). Three studies restricted recruitment to men who have sex with men (MSM) [12, 19, 24] and four specifically stated they only included adults.[17, 25, 26, 34] All other studies did not apply inclusion criteria, other than the study period.

Linkage to care by study and time from diagnosis can be seen in Table 2. However, the denominator used to calculate the linkage measure should be considered when making comparisons across studies. Over half of studies (59%; 13/22) measured the timeliness of linkage among those already established in care, excluding anyone with no care information available (e.g. excluding people missing CD4 count data). Within these 13, there were three studies which published linkage among all new HIV diagnoses but had to restrict estimates to those in care to examine the time between diagnosis and care entry.[13, 14, 21] As explained by authors that were contacted, this was most often due to incomplete date information.

**Table 2:** Linkage to HIV care at 2 weeks, 1 month, 3 months and 6 months after diagnosis: WHO European Region, 2006–2017 (n = 22 studies).

Author, year	Country of study	Linkage to care denominator	Linked to care within 2 weeks of diagnosis		Linked to care within 1 month of diagnosis		Linked to care within 3 months of diagnosis		Linked to care within 6 months of diagnosis	
			n	%	n	%	n	%	n	%
Chernyshev*‡‡, 2017 [24]	Ukraine	65	-	-	61	93.8%	-	-	-	-
del Campo*, 2016 [27]	Spain	112	-	-	71	63.4%	-	-	-	-
Chkhartishvili*, 2015 [25]	Georgia	1,563	-	-	-	-	1,229	78.6%	-	-
Raffo*, 2015 [29]	Spain	55	-	-	43	78.2%	50	90.9%	-	-
van Veen, 2015 [22]	Netherlands	259	-	-	215	83.0%	-	-	-	-
Zakowicz*, 2015 [32]	Russia, Ukraine, Georgia, Greece, Italy, Armenia, Ireland	Russia: 77; Other countries**: 61	-	-	-	-	Russia: 19; Other countries: 23	Russia: 24.7%; Other countries: 37.7%	-	-
Hall, 2013 [16]	Italy, Spain	Italy: 3,245; Spain: 1,519	-	-	-	-	Italy: 2,908; Spain: 1,154	Italy: 89.6%; Spain: 76.0%	-	-
Sprague*, 2011[33]	Estonia, Moldova, Poland, Turkey, and Ukraine	Estonia: 87; Moldova: 403; Poland: 504; Turkey: 100; Ukraine: 1,500	-	-	-	-	-	-	Estonia: 44; Moldova: 125; Poland: 292; Turkey: 90; Ukraine: 660	Estonia: 50.6%; Moldova: 31.0%; Poland: 57.9%; Turkey: 90.0%; Ukraine: 44.0%
Apea*, 2009 [23]	United Kingdom	88	82	93.2%	-	-	-	-	-	-
Croxford*, 2017 [26]	33 WHO European countries	60,139†	-	-	-	-	57,565	95.7%	-	-
Freeman-Romilly, 2017 [14]	United Kingdom	68†	-	-	61	89.7%	-	-	-	-
Elliot‡‡, 2016 [12]	United Kingdom	54†	-	-	51	94.4%	52	96.3%	-	-
Fernandez-Lopez, 2016 [13]	Denmark, Italy, Lithuania, Spain, Latvia	63†	-	-	-	-	63	100%	-	-
Girometti‡‡, 2017 [15]	United Kingdom	87†	-	-	-	-	83	95.4%	-	-
Kirwan*, 2016 [34]	United Kingdom	5,149†	3,856	74.9%	4,426	86.0%	4,981	96.7%	-	-
Kowalska, 2016 [18]	Poland	144†	-	-	99	68.8%	117	81.3%	-	-
Michie*, 2015 [28]	United Kingdom	64†	27	42.2%	-	-	-	-	-	-
Van Beckhoven, 2015 [21]	Belgium	3,523†‡	1,755	49.8%	2,497	70.9%	3,180	90.3%	-	-
Van Sighem*, 2015 [30]	Netherlands	858†	-	-	-	-	850	99.1%	-	-
Cuzin, 2013 [11]	France	2,670†	-	-	2,139	80.1%	2,311	86.6%	-	-
Kiriazova, 2013 [17]	Ukraine	6,101†	-	-	605	9.9%	2,894	47.4%	-	-
Meulbroek‡‡, 2013 [19]	Spain	448†	-	-	381	85.0%	-	-	-	-

* Conference proceedings or reports

**Countries combined with <50 diagnoses each

^†^ Number of people newly diagnosed in care

^‡^ Number of people that entered in care in the first year following diagnosis with a date of care

^‡‡^ MSM only

Nearly two thirds of the studies presented linkage to care within three months of diagnosis (63.6%; 14/22) (range: 25%-100%) (see meta-analysis below). Linkage to care within one month of diagnosis was described by over half of studies (54%; 12/22); eight measured the timeliness of linkage among those in care while the remaining four looked at linkage among all new HIV diagnoses. The proportion linked within a month among those in care ranged from 10% in a retrospective review on linkage among AIDS centre attendees in Ukraine [17] to 94% in a study of MSM diagnosed by HIV self-sampling in the UK.[12] The proportion of all new diagnoses linked within one month ranged from 63% in a study of people testing HIV-positive at a hospital in Spain [27] to 94% in a study of MSM undergoing community-based rapid testing in Ukraine.[24]

Only four studies presented linkage within two weeks, the majority from the UK and among those in care (range: 42%-93%).[21, 23, 28, 34] One cross-sectional study measured patient-reported linkage within six months (range: 31%-90%).[33] In the eight studies in which linkage was presented at multiple time intervals, linkage improved with time from diagnosis.

### Meta-analysis of linkage to care within three months

**Fig 2:**
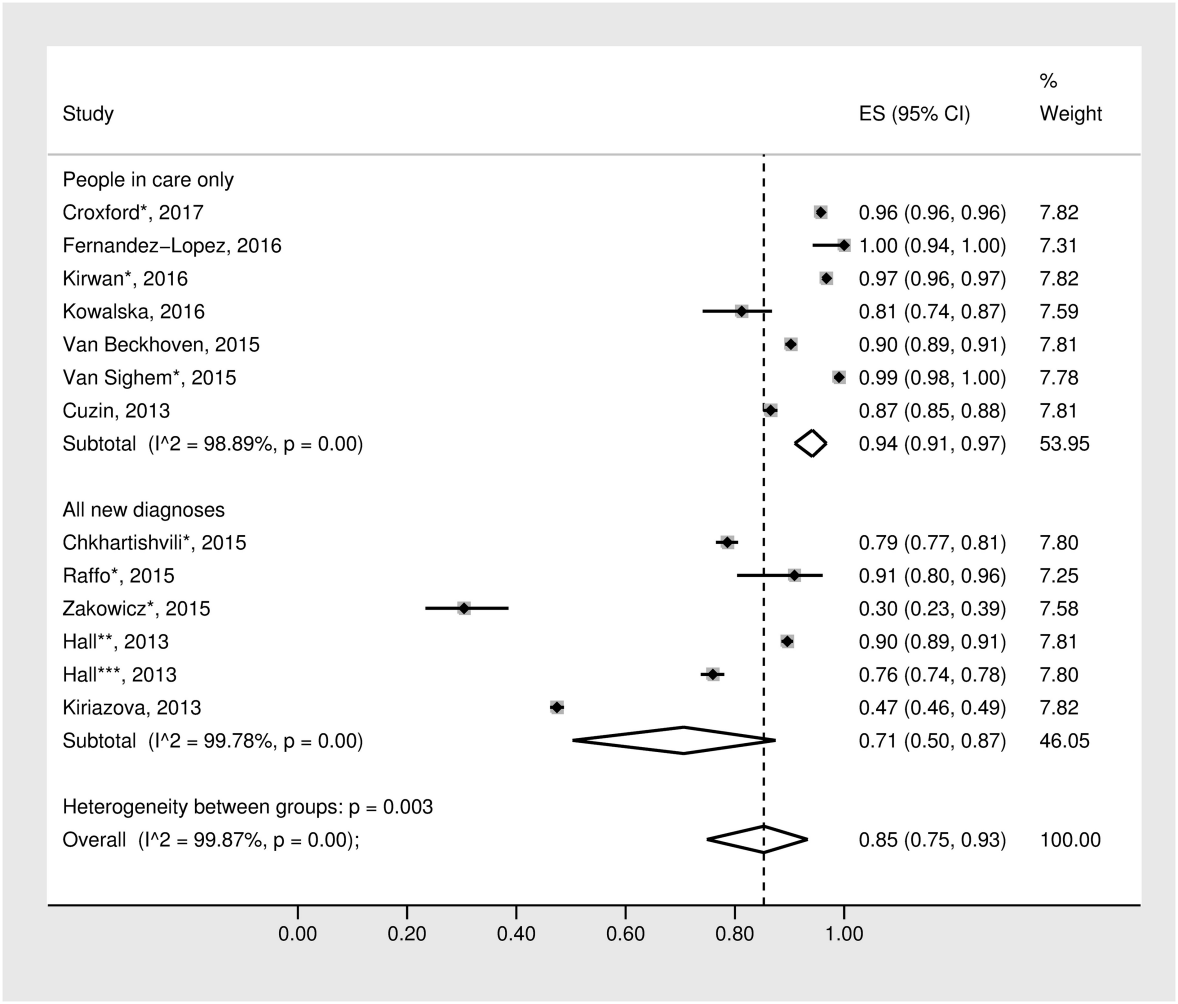
Forest plots for random effects meta-analysis of the proportion of people linked to care within three months of diagnosis by care status. *Conference proceedings or reports; **Hall, 2013—Italy; ***Hall, 2013—Spain.

The forest plot presenting the meta-analysis of the 12 studies that provided data on linkage to care at three months and weren’t restricted to MSM only can be seen in Fig 2.[11, 13, 16–18, 21, 25, 26, 29, 30, 32, 34] Six studies were published as peer-reviewed papers and six were reports or conference proceedings (not peer-reviewed). There was significant heterogeneity of results across studies (Q = 9451.38, I^2^ = 99.87%; p<0.001). The random-effects model generated a pooled estimate of 85% (95% CI: 75%-93%) linked to care within three months. This estimate increased slightly (92%, 95% CI: 88%–96%) after restricting analyses to the eight studies from countries covering the EU/European Economic Area (EEA).

To investigate potential sources of heterogeneity, the random effects meta-analysis was stratified by care status—whether each study described linkage to care among i) all new diagnoses of HIV or ii) those already in care. The aggregated estimate of the proportion of people linked within three months of those newly diagnosed with HIV was 71% (95% CI: 50%-87%) (Q = 2275.58, I^2^ = 99.78%; p<0.001), while the estimate for those already in care was as high as 94% (95% CI: 91%-97%) (Q = 539.32, I^2^ = 98.89%; p<0.001). The heterogeneity between these groups was significant (p = 0.003). In addition to heterogeneity between groups, heterogeneity within groups was also high (people in care: p<0.001; all new diagnoses p<0.001).

### Factors associated with linkage to care

There were seven studies that identified factors associated with linkage to care, the details of which can be seen in Table 1.[14, 18, 20–22, 26, 31] Meta-analysis was not deemed to be appropriate as there were a variety of outcomes examined (Table 3). While two studies investigated factors associated with being linked to care after diagnosis,[14, 18] the majority of studies looked at a negative outcome—either delayed entry into HIV care at one month or three months [20–22, 26, 31] or never having accessed care.[22] In addition, there were a number of different factors included in multivariable analysis and those that were similar across studies were not defined consistently (Table 3).

**Table 3:** Factors associated with linkage to care: WHO European region, 2006–2017 (n = 7 studies).

Author, year	Study design	Statistical analyses	Outcome	Adjustments in multivariable analysis			Risk factors for delayed linkage or not linking to care after diagnosis in multivariable analysis**
				Demographic factors	Diagnosis/clinical factors	Social/behavioural factors	
Croxford*†, 2017 [26]	Cohort	Logistic regression (OR)	Delayed linkage to care (>3 months after diagnosis)	Sex Age at diagnosis Region of origin	Diagnosis year Region of diagnosis Risk group First CD4 count after diagnosis	-	Being diagnosed in Central and Eastern Europe Acquiring HIV through heterosexual transmission, injecting drug use or other routes Being diagnosed in earlier years (pre-2012) Younger age at diagnosis (<55 years) Higher CD4 counts
Freeman-Romilly, 2017 [14]	Cohort	Logistic regression (OR)	Presenting for follow-up after diagnosis	Sex Age at test Sexual orientation Ethnicity	-	-	Acquiring HIV through heterosexual transmission
Kowalska, 2016 [18]	Cohort	Cox proportional hazards (HR)	Being linked to care after diagnosis	Age at test Sexual orientation	-	Education Partner HIV status Stable relationship status Condom use with stable partners	Bi/heterosexual sexual orientation Having lower levels of education Not using condoms with stable partners Younger age at test
Neduzhko†, 2016 [20]	Cross-sectional	Logistic regression (OR)	Delayed HIV care entry (>3 months after diagnosis)	-	Test location	Education Feeling ill Lack of time to attend for care	Not having time to go to the AIDS centre Not feeling ill at diagnosis Not having finished high school/high school/vocational school
Van Beckhoven, 2015 [21]	Cohort	Logistic regression (OR)	Not entering care within one year of diagnosis	Sex Age at diagnosis Nationality	Risk group Reason for testing	-	Testing for preoperative reasons Being of non-Belgian nationality (in Belgium)
van Veen, 2015 [22]	Cohort	Logistic regression (OR)	Not being linked to care within 4 weeks of diagnosis	Age at diagnosis Ethnicity	CD4 count at diagnosis Viral load at diagnosis Referral to care pathway	Insurance Steady relationship status HIV disclosure status	Being referred to care indirectly through general practice or self-referral Younger age at diagnosis (<25 years)
			Not linking to care after diagnosis	Age at diagnosis Ethnicity	Viral load at diagnosis Referral to care pathway	Insurance Previous HIV testing	Being referred to care indirectly through general practice or self-referral Having an undetectable viral load at diagnosis Lacking health insurance
Yin*†, 2012 [31]	Cohort	Logistic regression (OR)	Delayed baseline assessment (>1 month after diagnosis)	Sex Ethnicity	Risk group Geography of diagnosis Test location	-	Being diagnosed in general practice or other medical settings Acquiring HIV through injecting drug use Being diagnosed in the UK outside London

* Conference proceedings or reports

**In order of descending magnitude where possible

^†^ Among people in care only

OR = odds ratio; HR = hazard ratio

Factors found to be associated with delayed or not linking to care in multiple studies included (Table 3): acquiring HIV through heterosexual contact [14, 18, 26] or injecting drug use,[26, 31] being of younger age at diagnosis,[18, 22, 26] having lower levels of education,[18, 20] being or feeling well at diagnosis [20, 22, 26] and being diagnosed outside an STI clinic [22, 31].

### Quality assessment of included studies

Of the twelve peer-reviewed articles that could be quality assessed, most had limited generalisability, as they targeted specific high-risk populations such as MSM or people who inject drugs (PWID) accessing particular testing services (e.g. STI clinics, CBVCTs, etc.). A number of studies carried out a retrospective review of linkage among people already accessing HIV care, in which case findings may not be generalisable to individuals not in care. Selection bias was introduced in a few studies that recruited people from a selection of clinics or locations but there was no information provided on how the selected sites compared to ones not included. For those studies that reported on behavioural factors associated with linkage to care, many utilised self-reported behavioural data, potentially subjecting the results to social-desirability bias.

Overall, the quality of reporting was high. However, seven of the eleven cohort studies did not report on the length of follow-up. This was of particular concern for people diagnosed near the end of the study period; it was not clear if they had enough follow-up time for linkage to occur. A few studies were not clear on a definition of linkage to care but this was clarified after contact with the authors.

There were few methodological or statistical issues identified. Where described, missing data within each study was minimal and the risk of bias low. Full details of the quality assessment can be seen in Tables A and B in S2 Appendix.

## Discussion

In this systematic review, 24 studies were identified that used a standardised definition to measure linkage to care following HIV diagnosis since 2006 in the WHO European Region. Twenty-two studies provided an independent point estimate of linkage to care, with 14 studies measuring prompt linkage within three months. Seven studies addressed factors associated with not linking to care or delayed linkage.

Despite restricting inclusion to studies utilising a standard definition, the ability to compare estimates of linkage to care between included studies was limited by the varied populations and settings in which the studies were conducted, as well as substantial methodological differences, which created challenges in data synthesis and the interpretation of findings. Although generally, linkage tended to be lower in studies from countries with HIV epidemics driven by injecting drug use. Studies also used different time points from diagnosis to quantify prompt linkage to care—two weeks, one month, three months and six months.

Meta-analysis was restricted to 12 studies measuring prompt linkage at three months, producing a relatively high pooled estimate of 85% (95% CI: 75%-93%). This European estimate is similar to linkage figures from other Western countries, including Canada: 73%, the United States (US): 84% and Australia: 90%.[16, 35] Though, when data among those already in care were excluded, our pooled estimate of linkage among new diagnoses dropped to 71% (95% CI: 50%-87%). This is lower than estimates from Western countries outside of Europe; however, it is based on only six studies with high heterogeneity as evident by the wide confidence interval. In addition, half of the studies included are from Eastern Europe, in which almost half of new diagnoses each year are among PWID.[9] PWID are known to delay access to medical care, which can be attributed to a variety of social-environmental challenges, such as homelessness, and a lack of financial and psychosocial support.[26, 31, 36]

The pooled estimate of prompt linkage to care presented in this review must be interpreted with caution as heterogeneity was high (>99%). This heterogeneity between studies was partially explained through stratification by care status—separating studies into those that described linkage to care among everyone newly diagnosed and people already in care. Retrospective studies measuring timeliness of linkage among those established in care inflated linkage figures, as those who never entered HIV care were excluded. However, analyses showed heterogeneity was also high within care strata. This may be as a result of the diverse health systems across Europe and country-specific legal and regulatory barriers that may impede entry into care.[37, 38] In some countries, access to HIV care and treatment may be dependent on immigration-status or sexual orientation.[37] Certain risk groups may delay attending for care as they may fear incarceration or judgement.[38] Country-specific treatment guidelines may also inhibit people accessing HIV care. Despite the existence of European guidelines produced by the European AIDS Clinical Society recommending immediate ART initiation after diagnosis,[39] over a third (36% 17/47) of countries in the WHO European Region had treatment restrictions in place based on CD4 count in 2016.[40] Individual factors associated with poor linkage after diagnosis identified in this review included: acquiring HIV through heterosexual contact or injecting drug use, being of younger age at diagnosis, lower education levels, being or feeling well at diagnosis and being diagnosed outside an STI clinic.[14, 18, 20, 22, 26, 31] Additional barriers identified in qualitative studies from Europe outside the scope of this review include: problems with language and communication, poor care infrastructure, dissatisfaction with quality of services and medical staff and concerns over confidentiality and HIV status disclosure.[41, 42]

There have been a number of strategies and approaches found to be effective in improving and promoting prompt linkage to care for people newly diagnosed with HIV, such as behavioural interventions, peer support, intensified post-test counselling by community health workers, integrated testing and care services and support for HIV disclosure.[43, 44] Communication technologies, such as text messaging and calls via mobile phones have been found to improve linkage and retention in care.[43, 44] Contact tracing has been found to effectively identify HIV cases and is a service that can promote partners’ early referral to care.[43, 45] Removing legal and regulatory barriers to ensure equal access to care can be complex and challenging. European case studies on innovative advocacy strategies can be found on the OptTEST website.[46]

The findings of this systematic review are strengthened by the robust methodology applied, following PRISMA systematic review guidelines (S3 Appendix). Furthermore, the review was not limited to published studies; over half of the records included were conference proceedings or reports, which minimised the impact of publication bias. However, this review is subject to a number of limitations. The included studies were of variable quality, with the conference proceedings and reports not having undergone peer review. Included studies also had significant heterogeneity; as such, there were challenges with assessing associations and evidence synthesis. Pooled estimates should be interpreted with caution. Despite removing studies with duplicate data sources during screening, there may be some overlap and individuals that are included in more than one study. We estimate this may affect about 3% of the 89,006 individuals included in this review, based on the countries covered and the overlap of dates with the European estimate.[26] However, this upper estimate assumes national coverage of HIV surveillance programmes, including diagnoses in CBVCT and that all data are reported to European surveillance.

Another limitation is the geographical coverage of this review. Even though multiple databases were systematically searched to minimise bias, the searches only identified studies from 19 of the 53 countries in the WHO European Region. This may be due to the fact the review was restricted to papers published in English or a lack of published data from these regions. Data used to measure linkage to care are often not captured due to limited national and local surveillance and restrictions as to what information is able to be collected.[47] Finally, this review very much focussed on attendance to HIV specialist medical care after diagnosis. Those not in HIV care, that may have been accessing other services related to their HIV such as urgent care or may have died shortly after diagnosis, were not captured.

Attendance to an HIV care provider promptly after diagnosis is critical, as linkage to care facilitates access to HIV treatment. Immediate initiation of ART has substantial benefits, reducing the risk of patient morbidity and mortality, as well as reducing onward transmission.[1–3, 48] Ensuring prompt linkage to care for all people diagnosed with HIV is a key component in the effort to meet the UNAIDS 2020 targets to have 90% of people with HIV diagnosed, 90% on ART and 90% virally suppressed to end the AIDS epidemic by 2030.[49] In this systematic review, we present the most up-to-date evidence synthesis of research from Europe on linkage to care following HIV diagnosis. Overall, few countries in the WHO European Region have produced estimates on this essential HIV quality of care indicator. Where available, linkage estimates vary and reflect diverse health care systems, as well as political and socio-economic factors that may hinder people living with HIV from seeking care such as migrants and PWID. Further development of public health monitoring systems and adoption of a standard definition of prompt linkage are needed to monitor the equitable access to HIV care and treatment in the era of effective therapy. The OptTEST project advocates for a pragmatic approach to the public health monitoring of linkage to care using three months as the time cut-off.[6] This is in line with guidance from the ECDC, WHO and data published by the Centre for Disease Control in the US.[6, 8, 35]

## Supporting information

S1 Appendix Full systematic review database search strings. (DOCX)

S2 Appendix Quality assessment of included peer-reviewed articles. (DOCX)

S3 Appendix PRISMA checklist. (DOCX)
