# Supplementary material for: Linkage to HIV care following diagnosis in the WHO European Region: A systematic review and meta-analysis, 2006-2017
Source: PLoS One. 2018 Feb 16;13(2):e0192403. doi: 10.1371/journal.pone.0192403 (PMC5815583; doi:10.1371/journal.pone.0192403)
Supplement: S1 Appendix — (DOCX) [file pone.0192403.s001.docx]

S1 Appendix. Full systematic review database search strings

S1 Table A. Search strings for Ovid Medline (in-process & other non-indexed citations and Ovid MEDLINE)

| **#** | **Search string** |
| --- | --- |
| 1 | HIV/ |
| 2 | HIV-1/ |
| 3 | HIV-2/ |
| 4 | HIV Infections/ |
| 5 | (HIV or HIV infect* or HIV patient or HIV 1 or HIV 2 or HIV 1 infect* or HIV 2 infect* or human immunodeficiency virus or human immunodeficiency virus 1 or human immunodeficiency virus 1 infect* or human immunodeficiency virus 2 or human immunodeficiency virus 2 infect* or human immunodeficiency virus infect*).ab,ti. |
| 6 | Acquired Immunodeficiency Syndrome/ |
| 7 | (Acquired immune deficiency syndrome or acquired immunodeficiency syndrome or AIDS).ab,ti. |
| 8 | 1 or 2 or 3 or 4 or 5 or 6 or 7 |
| 9 | "Referral and Consultation"/ |
| 10 | Health Services Accessibility/ |
| 11 | "Quality of Health Care"/ |
| 12 | Quality Indicators, Health Care/ |
| 13 | "Standard of Care"/ |
| 14 | (care adj3 (link* or enrol* or consult* or access* or engag* or connect* or enter or enters or entered or entering or entry or entrance or initiat* or integrat* or attend* or quality or diagnosis)).ab,ti. |
| 15 | "Continuity of Patient Care"/ |
| 16 | (care adj3 (continuum or cascade*)).ab,ti. |
| 17 | "treatment cascade*".ab,ti. |
| 18 | 9 or 10 or 11 or 12 or 13 or 14 or 15 or 16 or 17 |
| 19 | exp Europe/ |
| 20 | exp Asia, Central/ or exp Asia, Northern/ |
| 21 | (Europe* or "Central Asia" or "Northern Asia" or Albania* or Armenia* or Andorra* or Austria* or Azerbaijan* or Belarus* or Belgium* or Belgian* or Bosnia* or Bulgaria* or Croatia* or Cyprus* or Cypriot* or Czech* or Denmark* or Danish* or Estonia* or Finland* or Finnish* or France* or French* or Georgia* or German* or Greece* or Greek* or Hungar* or Iceland* or Ireland* or Irish* or Israel* or Italy* or Italian* or Kazakhstan* or Kyrgyzstan* or Latvia* or Lithuania* or Malta* or Maltese* or Monaco* or Montenegro* or Netherlands* or Holland* or Dutch* or Norway* or Norwegian* or Poland* or Polish* or Portugal* or Portuguese* or Moldova* or Romania* or Russia* or "San Marino*" or Serbia* or Slovakia* or Spain* or Spanish* or Catalonia* or Sweden* or Swedish* or Swiss* or Switzerland* or Tajikistan* or Macedonia* or Turkey* or Turkish* or Turkmenistan* or Ukraine* or UK* or "United Kingdom*" or GB* or Britain* or British* or England* or Scotland* or Scottish* or Wales* or Welsh* or London* or Uzbekistan*).ab,kw,ti. |
| 22 | 19 or 20 or 21 |
| 23 | 8 and 18 and 22 |
| 24 | limit 23 to (english language and yr="2006 -Current") |

 S1 Table B. Search strings for Embase

| **[#](http://ovidsp.tx.ovid.com.libproxy.ucl.ac.uk/sp-3.18.0b/ovidweb.cgi?&S=GOANFPDBEDDDJDMENCJKPHDCFEILAA00&Sort+Sets=descending)** | **Search string** |
| --- | --- |
| 1 | Human immunodeficiency virus/ |
| 2 | Human immunodeficiency virus 1/ |
| 3 | Human immunodeficiency virus 2/ |
| 4 | Human immunodeficiency virus infection/ |
| 5 | Human immunodeficiency virus 1 infection/ |
| 6 | Human immunodeficiency virus 2 infection/ |
| 7 | Human immunodeficiency virus infected patient/ |
| 8 | (HIV or HIV infect* or HIV patient or HIV 1 or HIV 2 or HIV 1 infect* or HIV 2 infect* or human immunodeficiency virus or human immunodeficiency virus 1 or human immunodeficiency virus 1 infect* or human immunodeficiency virus 2 or human immunodeficiency virus 2 infect* or human immunodeficiency virus infect*).ti,ab. |
| 9 | 1 or 2 or 3 or 4 or 5 or 6 or 7 or 8 |
| 10 | acquired immune deficiency syndrome/ |
| 11 | AIDS patient/ |
| 12 | (Acquired immune deficiency syndrome or acquired immunodeficiency syndrome or AIDS).ti,ab. |
| 13 | 10 or 11 or 12 |
| 14 | 9 or 13 |
| 15 | patient referral/ |
| 16 | health care access/ |
| 17 | health care quality/ |
| 18 | patient assessment/ |
| 19 | (care adj3 (link* or enrol* or consult* or access* or engag* or connect* or enter or enters or entered or entering or entry or entrance or initiat* or integrat* or attend* or quality or diagnosis)).ti,ab. |
| 20 | patient care/ |
| 21 | (care adj3 (continuum or cascade*)).ti,ab. |
| 22 | "treatment cascade*".ti,ab. |
| 23 | 15 or 16 or 17 or 18 or 19 or 20 or 21 or 22 |
| 24 | exp Europe/ |
| 25 | exp Asia/ |
| 26 | (Europe* or "Central Asia" or "Northern Asia" or Albania* or Armenia* or Andorra* or Austria* or Azerbaijan* or Belarus* or Belgium* or Belgian* or Bosnia* or Bulgaria* or Croatia* or Cyprus* or Cypriot* or Czech* or Denmark* or Danish* or Estonia* or Finland* or Finnish* or France* or French* or Georgia* or German* or Greece* or Greek* or Hungar* or Iceland* or Ireland* or Irish* or Israel* or Italy* or Italian* or Kazakhstan* or Kyrgyzstan* or Latvia* or Lithuania* or Malta* or Maltese* or Monaco* or Montenegro* or Netherlands* or Holland* or Dutch* or Norway* or Norwegian* or Poland* or Polish* or Portugal* or Portuguese* or Moldova* or Romania* or Russia* or "San Marino*" or Serbia* or Slovakia* or Spain* or Spanish* or Catalonia* or Sweden* or Swedish* or Swiss* or Switzerland* or Tajikistan* or Macedonia* or Turkey* or Turkish* or Turkmenistan* or Ukraine* or UK* or "United Kingdom*" or GB* or Britain* or British* or England* or Scotland* or Scottish* or Wales* or Welsh* or London* or Uzbekistan*).ti,ab,kw. |
| 27 | 24 or 25 or 26 |
| 28 | 14 and 23 and 27 |
| 29 | limit 28 to (english language and yr="2006 -Current") |

S1 Table C. Search string for PubMed

| **#** | **Search string** |
| --- | --- |
| 1 | HIV[MeSH Terms] OR HIV infections[MeSH Terms] |
| 2 | HIV[Title/Abstract] OR HIV infect*[Title/Abstract] OR HIV patient[Title/Abstract] OR HIV 1[Title/Abstract] OR HIV 2[Title/Abstract] OR HIV 1 infect*[Title/Abstract] OR HIV 2 infect*[Title/Abstract] OR human immunodeficiency virus[Title/Abstract] OR human immunodeficiency virus 1[Title/Abstract] OR human immunodeficiency virus 1 infect*[Title/Abstract] OR human immunodeficiency virus 2[Title/Abstract] OR human immunodeficiency virus 2 infect*[Title/Abstract] OR human immunodeficiency virus infect*[Title/Abstract] |
| 3 | Acquired Immunodeficiency Syndrome[MeSH Terms] |
| 4 | Acquired immune deficiency syndrome[Title/Abstract] OR acquired immunodeficiency syndrome[Title/Abstract] OR AIDS[Title/Abstract] |
| 5 | (#1 or #2 or #3 or #4) |
| 6 | "Continuity of Patient Care"[Mesh:NoExp] OR "Quality of Health Care"[Mesh:NoExp] OR "Quality Indicators, Health Care"[Mesh] OR "Standard of Care"[Mesh:NoExp] OR "Referral and Consultation"[Mesh:NoExp] OR "Health Services Accessibility"[Mesh:NoExp] |
| 7 | treatment cascade*[Title/Abstract] OR continuum* of care[Title/Abstract] OR care continuum*[Title/Abstract] OR care cascade*[Title/Abstract] OR cascade* of care[Title/Abstract] |
| 8 | care[Title/Abstract]) AND (link*[Title/Abstract] OR enrol*[Title/Abstract] OR consult*[Title/Abstract] OR access*[Title/Abstract] OR engag*[Title/Abstract] OR connect*[Title/Abstract] OR enter[Title/Abstract] OR enters[Title/Abstract] OR entered[Title/Abstract] OR entering[Title/Abstract] OR entry[Title/Abstract] OR entrance[Title/Abstract] OR initiat*[Title/Abstract] OR integrat*[Title/Abstract] OR attend*[Title/Abstract] OR quality[Title/Abstract] OR diagnosis[Title/Abstract]) |
| 9 | (#6 or #7 or #8) |
| 10 | Europe[MeSH Terms] |
| 11 | asia, central[MeSH Terms] OR asia, northern[MeSH Terms] |
| 12 | Europe*[Title/Abstract] OR "Central Asia"[Title/Abstract] OR "Northern Asia"[Title/Abstract] OR Albania*[Title/Abstract] OR Armenia*[Title/Abstract] OR Andorra*[Title/Abstract] OR Austria*[Title/Abstract] OR Azerbaijan*[Title/Abstract] OR Belarus*[Title/Abstract] OR Belgium*[Title/Abstract] OR Belgian*[Title/Abstract] OR Bosnia*[Title/Abstract] OR Bulgaria*[Title/Abstract] OR Croatia*[Title/Abstract] OR Cyprus*[Title/Abstract] OR Cypriot*[Title/Abstract] OR Czech*[Title/Abstract] OR Denmark*[Title/Abstract] OR Danish*[Title/Abstract] OR Estonia*[Title/Abstract] OR Finland*[Title/Abstract] OR Finnish*[Title/Abstract] OR France*[Title/Abstract] OR French*[Title/Abstract] OR Georgia*[Title/Abstract] OR German*[Title/Abstract] OR Greece*[Title/Abstract] OR Greek*[Title/Abstract] OR Hungar*[Title/Abstract] OR Iceland*[Title/Abstract] OR Ireland*[Title/Abstract] OR Irish*[Title/Abstract] OR Israel*[Title/Abstract] OR Italy*[Title/Abstract] OR Italian*[Title/Abstract] OR Kazakhstan*[Title/Abstract] OR Kyrgyzstan*[Title/Abstract] OR Latvia*[Title/Abstract] OR Lithuania*[Title/Abstract] OR Malta*[Title/Abstract] OR Maltese*[Title/Abstract] OR Monaco*[Title/Abstract] OR Montenegro*[Title/Abstract] OR Netherlands*[Title/Abstract] OR Holland*[Title/Abstract] OR Dutch*[Title/Abstract] OR Norway*[Title/Abstract] OR Norwegian*[Title/Abstract] OR Poland*[Title/Abstract] OR Polish*[Title/Abstract] OR Portugal*[Title/Abstract] OR Portuguese*[Title/Abstract] OR Moldova*[Title/Abstract] OR Romania*[Title/Abstract] OR Russia*[Title/Abstract] OR "San Marino*"[Title/Abstract] OR Serbia*[Title/Abstract] OR Slovakia*[Title/Abstract] OR Spain*[Title/Abstract] OR Spanish*[Title/Abstract] OR Catalonia*[Title/Abstract] OR Sweden*[Title/Abstract] OR Swedish*[Title/Abstract] OR Swiss*[Title/Abstract] OR Switzerland*[Title/Abstract] OR Tajikistan*[Title/Abstract] OR Macedonia*[Title/Abstract] OR Turkey*[Title/Abstract] OR Turkish*[Title/Abstract] OR Turkmenistan*[Title/Abstract] OR Ukraine*[Title/Abstract] OR UK[Title/Abstract] OR "United Kingdom*"[Title/Abstract] OR GB[Title/Abstract] OR Britain*[Title/Abstract] OR British*[Title/Abstract] OR England*[Title/Abstract] OR Scotland*[Title/Abstract] OR Scottish*[Title/Abstract] OR Wales*[Title/Abstract] OR Welsh*[Title/Abstract] OR London*[Title/Abstract] OR Uzbekistan*[Title/Abstract] |
| 13 | (#10 or #11 or #12) |
| 14 | (#5 AND #9 AND #13) |
| 15 | Publication date from 2006/01/01; English |

S1 Table D. Search strings for Web of Science

| **#** | **Search string** |
| --- | --- |
| 1 | (TS=("HIV" OR "HIV infect*" OR "HIV patient" OR "HIV 1" OR "HIV 2" OR "HIV 1 infect*" OR "HIV 2 infect*" OR "human immunodeficiency virus" OR "human immunodeficiency virus 1" OR "human immunodeficiency virus 1 infect*" OR "human immunodeficiency virus 2" OR "human immunodeficiency virus 2 infect*" OR "human immunodeficiency virus infect*")) *AND***LANGUAGE:** (English) Indexes=SCI-EXPANDED, SSCI, A&HCI, CPCI-S, CPCI-SSH, BKCI-S, ESCI, CCR-EXPANDED, IC Timespan=2006-2017 |
| 2 | (TS=("Acquired immune deficiency syndrome" OR "acquired immunodeficiency syndrome" OR "AIDS")) *AND***LANGUAGE:** (English) Indexes=SCI-EXPANDED, SSCI, A&HCI, CPCI-S, CPCI-SSH, BKCI-S, ESCI, CCR-EXPANDED, IC Timespan=2006-2017 |
| 3 | #2 OR #1 Indexes=SCI-EXPANDED, SSCI, A&HCI, CPCI-S, CPCI-SSH, BKCI-S, ESCI, CCR-EXPANDED, IC Timespan=2006-2017 |
| 4 | (TS=(care near/3 (link* or enrol* or consult* or access* or engag* or connect* or enter or enters or entered or entering or entry or entrance or initiat* or integrat* or attend* or quality or diagnosis))) *AND***LANGUAGE:** (English) Indexes=SCI-EXPANDED, SSCI, A&HCI, CPCI-S, CPCI-SSH, BKCI-S, ESCI, CCR-EXPANDED, IC Timespan=2006-2017 |
| 5 | (TS=(care near/3 (continuum or cascade*))) *AND***LANGUAGE:** (English) Indexes=SSCI, A&HCI, CPCI-S, CPCI-SSH, BKCI-S, ESCI, CCR-EXPANDED, IC Timespan=2006-2017 |
| 6 | (TS="treatment cascade*") *AND***LANGUAGE:** (English) Indexes=SSCI, A&HCI, CPCI-S, CPCI-SSH, BKCI-S, ESCI, CCR-EXPANDED, IC Timespan=2006-2017 |
| 7 | #6 OR #5 OR #4 Indexes=SSCI, A&HCI, CPCI-S, CPCI-SSH, BKCI-S, ESCI, CCR-EXPANDED, IC Timespan=2006-2017 |
| 8 | (TS=("Europe*" or "Central Asia" or "Northern Asia" or "Albania*" or "Armenia*" or "Andorra*" or "Austria*" or "Azerbaijan*" or "Belarus*" or "Belgium*" or "Belgian*" or "Bosnia*" or "Bulgaria*" or "Croatia*" or "Cyprus*" or "Cypriot*" or "Czech*" or "Denmark*" or "Danish*" or "Estonia*" or "Finland*" or "Finnish*" or "France*" or "French*" or "Georgia*" or "German*" or "Greece*" or "Greek*" or "Hungar*" or "Iceland*" or "Ireland*" or "Irish*" or "Israel*" or "Italy*" or "Italian*" or "Kazakhstan*" or "Kyrgyzstan*" or "Latvia*" or "Lithuania*" or "Malta*" or "Maltese*" or "Monaco*" or "Montenegro*" or "Netherlands*" or "Holland*" or "Dutch*" or "Norway*" or "Norwegian*" or "Poland*" or "Polish*" or "Portugal*" or "Portuguese*" or "Moldova*" or "Romania*" or "Russia*" or "San Marino*" or "Serbia*" or "Slovakia*" or "Spain*" or "Spanish*" or "Catalonia*" or "Sweden*" or "Swedish*" or "Swiss*" or "Switzerland*" or "Tajikistan*" or "Macedonia*" or "Turkey*" or "Turkish*" or "Turkmenistan*" or "Ukraine*" or "UK" or "United Kingdom*" or "GB" or "Britain*" or "British*" or "England*" or "Scotland*" or "Scottish*" or "Wales*" or "Welsh*" or "London*" or "Uzbekistan*")) *AND***LANGUAGE:** (English) Indexes=SSCI, A&HCI, CPCI-S, CPCI-SSH, BKCI-S, ESCI, CCR-EXPANDED, IC Timespan=2006-2017 |
| 9 | #8 AND #7 AND #3 Indexes=SSCI, A&HCI, CPCI-S, CPCI-SSH, BKCI-S, ESCI, CCR-EXPANDED, IC Timespan=2006-2017 |

S1 Table E. Search terms for the Cochrane Library

| **#** | **Search string** |
| --- | --- |
| 1 | MeSH descriptor: [HIV] explode all trees |
| 2 | MeSH descriptor: [Acquired Immunodeficiency Syndrome] explode all trees |
| 3 | "HIV" or "HIV infect*" or "HIV patient" or "HIV 1" or "HIV 2" or "HIV 1 infect*" or "HIV 2 infect*" or "human immunodeficiency virus" or "human immunodeficiency virus 1" or "human immunodeficiency virus 1 infect*" or "human immunodeficiency virus 2" or "human immunodeficiency virus 2 infect*" or "human immunodeficiency virus infect*":ti,ab,kw (Word variations have been searched) |
| 4 | "Acquired immune deficiency syndrome" or "acquired immunodeficiency syndrome" or "AIDS":ti,ab,kw (Word variations have been searched) |
| 5 | MeSH descriptor: [Continuity of Patient Care] this term only |
| 6 | MeSH descriptor: [Quality of Health Care] this term only |
| 7 | MeSH descriptor: [Quality Indicators, Health Care] explode all trees |
| 8 | MeSH descriptor: [Standard of Care] this term only |
| 9 | MeSH descriptor: [Referral and Consultation] this term ony |
| 10 | MeSH descriptor: [Health Services Accessibility] explode all trees |
| 11 | “treatment cascade*" or "continuum* of care" or "care continuum*" or "care cascade*" or "cascade* of care":ti,ab,kw (Word variations have been searched) |
| 12 | care and (link* or enrol* or consult* or access* or engag* or connect* or enter or enters or entered or entering or entry or entrance or initiat* or integrat* or attend* or quality or diagnosis):ti,ab,kw (Word variations have been searched) |
| 13 | MeSH descriptor: [Europe] explode all trees |
| 14 | MeSH descriptor: [Asia, Northern] explode all trees |
| 15 | MeSH descriptor: [Asia, Central] explode all trees |
| 16 | Europe* or "Central Asia" or "Northern Asia" or Albania* or Armenia* or Andorra* or Austria* or Azerbaijan* or Belarus* or Belgium* or Belgian* or Bosnia* or Bulgaria* or Croatia* or Cyprus* or Cypriot* or Czech* or Denmark* or Danish* or Estonia* or Finland* or Finnish* or France* or French* or Georgia* or German* or Greece* or Greek* or Hungar* or Iceland* or Ireland* or Irish* or Israel* or Italy* or Italian* or Kazakhstan* or Kyrgyzstan* or Latvia* or Lithuania* or Malta* or Maltese* or Monaco* or Montenegro* or Netherlands* or Holland* or Dutch* or Norway* or Norwegian* or Poland* or Polish* or Portugal* or Portuguese* or Moldova* or Romania* or Russia* or "San Marino*" or Serbia* or Slovakia* or Spain* or Spanish* or Catalonia* or Sweden* or Swedish* or Swiss* or Switzerland* or Tajikistan* or Macedonia* or Turkey* or Turkish* or Turkmenistan* or Ukraine* or UK or "United Kingdom*" or GB or Britain* or British* or England* or Scotland* or Scottish* or Wales* or Welsh* or London* or Uzbekistan*:ti,ab,kw (Word variations have been searched) |
| 17 | #1 or #2 or #3 or #4 |
| 18 | #5 or #6 or #7 or #8 or #9 or #10 or #11 or #12 |
| 19 | #13 or #14 or #15 or #16 |
| 20 | #17 and #18 and #19 Publication Year from 2006 to 2017 |

S1 Table F. Search strings for PsycINFO

| **#** | **Search string** |
| --- | --- |
| 1 | HIV/ |
| 2 | (HIV or HIV infect* or HIV patient or HIV 1 or HIV 2 or HIV 1 infect* or HIV 2 infect* or human immunodeficiency virus or human immunodeficiency virus 1 or human immunodeficiency virus 1 infect* or human immunodeficiency virus 2 or human immunodeficiency virus 2 infect* or human immunodeficiency virus infect*).ab,ti. |
| 3 | AIDS/ |
| 4 | (Acquired immune deficiency syndrome or acquired immunodeficiency syndrome or AIDS).ab,ti. |
| 5 | 1 or 2 or 3 or 4 |
| 6 | "Quality of Care"/ |
| 7 | "continuum of care"/ |
| 8 | (care adj3 (link* or enrol* or consult* or access* or engag* or connect* or enter or enters or entered or entering or entry or entrance or initiat* or integrat* or attend* or quality or diagnosis)).ab,ti. |
| 9 | (care adj3 (continuum or cascade*)).ab,ti. |
| 10 | "treatment cascade*".ab,ti. |
| 11 | 6 or 7 or 8 or 9 or 10 |
| 12 | (Europe* or "Central Asia" or "Northern Asia" or Albania* or Armenia* or Andorra* or Austria* or Azerbaijan* or Belarus* or Belgium* or Belgian* or Bosnia* or Bulgaria* or Croatia* or Cyprus* or Cypriot* or Czech* or Denmark* or Danish* or Estonia* or Finland* or Finnish* or France* or French* or Georgia* or German* or Greece* or Greek* or Hungar* or Iceland* or Ireland* or Irish* or Israel* or Italy* or Italian* or Kazakhstan* or Kyrgyzstan* or Latvia* or Lithuania* or Malta* or Maltese* or Monaco* or Montenegro* or Netherlands* or Holland* or Dutch* or Norway* or Norwegian* or Poland* or Polish* or Portugal* or Portuguese* or Moldova* or Romania* or Russia* or "San Marino*" or Serbia* or Slovakia* or Spain* or Spanish* or Catalonia* or Sweden* or Swedish* or Swiss* or Switzerland* or Tajikistan* or Macedonia* or Turkey* or Turkish* or Turkmenistan* or Ukraine* or UK* or "United Kingdom*" or GB* or Britain* or British* or England* or Scotland* or Scottish* or Wales* or Welsh* or London* or Uzbekistan*).ab,ti. |
| 13 | 5 and 11 and 12 |
| 14 | limit 13 to (english language and yr="2006 -Current") |
