# Supplementary material for: Linkage to HIV care following diagnosis in the WHO European Region: A systematic review and meta-analysis, 2006-2017
Source: PLoS One. 2018 Feb 16;13(2):e0192403. doi: 10.1371/journal.pone.0192403 (PMC5815583; doi:10.1371/journal.pone.0192403)
Supplement: S2 Appendix — (DOCX) [file pone.0192403.s002.docx]

S2 Appendix. Quality assessment of included peer-reviewed articles

S2 Table A. Detailed quality assessments of peer-reviewed studies

| **Quality assessment** | | **Freeman-Romilly, 2017 [18]** | **Girometti, 2017**  **[15]** | **Elliot, 2016**  **[12]** | **Fernandez-Lopez, 2016 [13]** | **Kowalska, 2016**  **[18]** | **Neduzhko, 2016**  **[20]** |
| --- | --- | --- | --- | --- | --- | --- | --- |
| Study design | | Cohort | Cohort | Cohort | Cohort | Cohort | Cross-sectional |
| All studies | Were the aims/ objectives of the study clear? | Yes | Yes | Yes | Yes | Yes | Yes |
|  | Was the study design appropriate for the stated aim? | Yes | Yes | Yes | Yes | Yes | Yes |
|  | Were the methods sufficiently described? | No | Yes | Yes | No | Yes | Yes |
|  | Were the risk factors/ outcomes measured correctly? | Unclear | Yes | Yes | Yes | Yes | Yes |
|  | Were the basic data adequately described? | Yes | Yes | Yes | Yes | Yes | No |
|  | Was the study population clearly defined? | Yes | Yes | Yes | No | Yes | Yes |
|  | Were results for analyses described in the methods presented? | Yes | Yes | Yes | Yes | Yes | Yes |
|  | Is it clear what was used to determine statistical significance? | Yes | Yes | NA | NA | Yes | Yes |
|  | Were the results internally consistent? | Yes | Yes | Yes | Yes | Yes | Yes |
|  | Were the risk factors/outcomes measured appropriate to the aims? | Unclear | Yes | Yes | Yes | Yes | Yes |
|  | Were the discussion/conclusions justified by the results? | Yes | Yes | Yes | Yes | Yes | Yes |
|  | Were the limitations discussed? | Yes | Yes | Yes | Yes | Yes | Yes |
|  | Were there any funding or conflicts of interest that may affect result interpretation? | No | No | No | No | No | No |
|  | Was ethical approval attained? | Yes | NA | NA | Unclear | Yes | Unclear |
|  | Could the study be replicable in other populations? | Yes | Yes | Yes | Yes | Yes | Yes |
| Cohort studies | Was the sample size justified? | NA | NA | NA | Yes | NA | - |
|  | Was follow-up described? | No | No | No | No | Yes | - |
|  | Was follow-up long enough for outcomes to occur? | Unclear | Unclear | Unclear | Unclear | Yes | - |
|  | Was the selection process likely to select representative sample? | Unclear | Yes | Yes | Yes | Yes | - |
|  | Did the study use a precise definition of the outcome? | No | Yes | Yes | Yes | Yes | - |
| Cross sectional studies | Was selection process likely to select representative sample? | - | - | - | - | - | Yes |
|  | Were measures taken to address/categorise non-responders? | - | - | - | - | - | No |
|  | Does the response rate raise concerns about non-response bias? | - | - | - | - | - | No |
|  | Was the sample size justified? | - | - | - | - | - | Yes |
| All studies | Generalisability and risk of bias | - Missing data impacts the validity of the study  - Limited generalisability - no information on THT attendees compare to other community service users  - Incomplete and delayed reporting - PHE did not have all the clinic baseline CD4 cell counts  - Follow-up period not defined so possible censorship bias | - Limited generalisability for non-MSM and outside STI clinic diagnosis setting - even though everyone recruited, only MSM took part  - Selection bias - one clinic in London, specific population attending, ART regimen chosen partly chosen according to clinician judgement - biased outcome on this possible | - Limited generalisability - Intervention only targets men who go online for sex, this group may participate in sexual activity that puts them at a different risk of HIV to others  - Selection bias - service relied on MSM having an address to mail self-sampling kit | - Limited generalisability - only selection of CBVCTs from the COBATEST network so results are not generalizable to all CBVCTs in Europe, and cannot be representative at the national or European level, no information on testing offered/accepted | - Limited generalisability - majority of study population MSM, which may limit ability to form conclusions on other risk groups due to small sample sizes  - Selection bias - no information on the three community clinics included  - Social desirability bias - self-reported risk behaviours | - Limited generalisability - clinic-based sample of people who eventually entered HIV care, findings may not be generalizable for those completely disconnected from health care system who may never seek HIV care, no information on people who did not participate  - Selection bias - data from only one region included only but no information on this region  - Social desirability bias - self-reported diagnosis date and risk behaviours |
|  | Quality of reporting | - Data categories different in multivariable than in descriptive analysis  - No information presented for those who did not link to care  - Follow-up period not defined  - Full data for regression not provided | No concerns | - No information on how data on confirmatory testing or linkage to care obtained | - Missing data not presented | - Missing data not presented | - No descriptive data for two variables included in the multivariable models  - No information on non-responders |
|  | Statistical issues | - No presentation of univariate analysis  - Unclear number included in multivariable analysis  - Level of significance not specified | No concerns | No concerns | No concerns | No concerns | No concerns |

S2 Table B. Detailed quality assessments of peer-reviewed studies continued

| **Quality assessment** | | **Van Beckhoven, 2015 [21]** | **van Veen, 2015**  **[22]** | **Cuzin, 2013**  **[11]** | **Hall, 2013**  **[16]** | **Kiriazova, 2013**  **[17]** | **Meulbroek, 2013 [19]** |
| --- | --- | --- | --- | --- | --- | --- | --- |
| Study design | | Cohort | Cohort | Cohort | Cohort | Cohort | Cohort |
| All studies | Were the aims/ objectives of the study clear? | Yes | Yes | Yes | Yes | Yes | Yes |
|  | Was the study design appropriate for the stated aim? | Yes | Yes | Yes | Yes | Yes | Yes |
|  | Were the methods sufficiently described? | Yes | Yes | No | Yes | Yes | No |
|  | Were the risk factors/ outcomes measured correctly? | Yes | Yes | Yes | Yes | Yes | Yes |
|  | Were the basic data adequately described? | Yes | Yes | Yes | Yes | No | No |
|  | Was the study population clearly defined? | Yes | Yes | Yes | Yes | Yes | No |
|  | Were results for analyses described in the methods presented? | Yes | Yes | Yes | Yes | No | Yes |
|  | Is it clear what was used to determine statistical significance? | No | Yes | NA | NA | Yes | NA |
|  | Were the results internally consistent? | Yes | Yes | Unclear | Yes | Unclear | Yes |
|  | Were the risk factors/outcomes measured appropriate to the aims? | Yes | No | Yes | Yes | Yes | Yes |
|  | Were the discussion/conclusions justified by the results? | Yes | No | Yes | Yes | Unclear | Yes |
|  | Were the limitations discussed? | Yes | Yes | Yes | Yes | Yes | No |
|  | Were there any funding or conflicts of interest that may affect result interpretation? | No | No | No | Unclear | No | Unclear |
|  | Was ethical approval attained? | Yes | Yes | NA | Unclear | Unclear | Unclear |
|  | Could the study be replicable in other populations? | Yes | Yes | No | Yes | Yes | Yes |
| Cohort studies | Was the sample size justified? | NA | NA | Yes | Yes | NA | NA |
|  | Was follow-up described? | Yes | Yes | No | Yes | No | No |
|  | Was follow-up long enough for outcomes to occur? | Yes | Yes | Unclear | Yes | Unclear | Unclear |
|  | Was the selection process likely to select representative sample? | Yes | Yes | Yes | Yes | Yes | NA |
|  | Did the study use a precise definition of the outcome? | Yes | Yes | No | Yes | Yes | Yes |
| All studies | Generalisability and risk of bias | No concerns | - Limited generalisability to non-STI clinic attendees, non-MSM populations and to people in other countries that don't need health insurance to access care.  - Social desirability bias - self-reported risk behaviours | - Limited generalisability - only includes those already in care  - Legal issues with directly link HIV diagnosis and entry in care. It could be possible that analysing very distinct populations, if at the extreme all people living in 1 region used to seek care elsewhere. | - Limited generalisability - study does not cover the entirety of each country with regions missing  - Selection bias - not complete coverage of surveillance and no info on those not included | - Limited generalisability - clinic-based sample of people who eventually entered HIV care, findings may not be generalizable for those completely disconnected from health care system who may never seek HIV care, no information on people who did not participate  - Selection bias - data from only one region included only but no information on this region | - Limited generalisability - to MSM not attending CBVCT  - Not able to assess selection bias as no baseline data collected on MSM tested (age etc.) |
|  | Quality of reporting | - Proportions presented for univariate analyses for some variables (unknown numerators/denominators) | - Missing data not reported for all variables  - No information on 30% of people who did not respond | No concerns | - Unclear as to what year of data was presented  - Incomplete reporting of test results may have underestimated linkage to care | - No data describing patient characteristics even though authors report using data on age and residency for analysis | - No justification as to why linkage to care only able to be measured 2009 onwards |
|  | Statistical issues | - Level of significance not specified  - No description of multivariable analysis in methods | - Small numbers in comparison in Table 1 | No concerns | No concerns | - No results of statistical tests provided | No concerns |
